# Utilization of research findings for health policy making and practice: evidence from three case studies in Bangladesh

**DOI:** 10.1186/s12961-015-0015-x

**Published:** 2015-05-28

**Authors:** David Roger Walugembe, Suzanne N. Kiwanuka, Joseph K. B. Matovu, Elizeus Rutebemberwa, Laura Reichenbach

**Affiliations:** Makerere University College of Health Sciences, School of Public Health, P.O. Box 7072, Kampala, Uganda; International Centre for Diarrhoeal Disease Research (icddr,b), P.O.Box 128, Dhaka, 1000 Bangladesh

**Keywords:** Evidence, Health, Policymaking, Research uptake, Research utilization

## Abstract

**Background:**

In striving to contribute towards improved health outcomes, health research institutions generate and accumulate huge volumes of relevant but often underutilized data. This study explores activities undertaken by researchers from the International Centre for Diarrhoeal Disease Research, Bangladesh (icddr,b), an international research institution that promotes the utilization of their findings in the policymaking processes in Bangladesh.

**Methods:**

The study used an exploratory case study design and employed qualitative methods to explore activities implemented to promote research utilization and the extent to which researchers felt that their findings contributed to the policymaking process. Data were collected between September and December 2011 through key informant interviews, focus group discussions with study investigators, and database and document reviews. We reviewed findings from 19 reproductive health studies conducted and completed by icddr,b researchers between 2001 and 2011. We interviewed 21 key informants, including 13 researchers, two policy makers, and six programme implementers. Data were entered into Microsoft Word and analyzed manually following a thematic framework approach. Following the World Health Organization/Turning Research into Practice (WHO/TRIP) framework, three case studies of how research findings were utilized in the policymaking processes in Bangladesh were documented.

**Results:**

Activities implemented to promote research utilization included conducting dissemination workshops, publishing scientific papers, developing policy briefs, providing technical assistance to policymakers and programme implementers, holding one-on-one meetings, and joining advocacy networks. The majority of the researchers (12 of 13) reported that their study findings were utilized to influence policymaking processes at different levels. However, some researchers reported being unaware of whether and how their findings were utilized. As regards actual utilization of research findings, the evidence from the three case studies indicate that research findings can be utilized instrumentally, conceptually and symbolically, and at different stages within the policymaking process, including agenda setting and policy formulation and implementation.

**Conclusions:**

The results show that research findings from icddr,b were promoted and utilized in health policymaking processes in Bangladesh using a variety of utilization approaches. These results suggest a need for using multiple approaches to promote utilization of research findings in health policymaking processes.

## Background

Research utilization refers to “making decisions concerning policy, advocacy and resource allocation, planning and management, and program systems development and strengthening, using information generated from research” [1]. The concept of research utilization has been described using a variety of terminologies, including ‘knowledge translation’, ‘knowledge management’, ‘knowledge utilization’, and ‘research dissemination’, among others. However, research utilization differs from all these terminologies in that it focuses on purposes and impact of the study rather than mere knowledge management or dissemination of results. More specifically, research utilization focuses on what researchers want people to receive from their research results, how they want people to make use of the ideas, information, or products resulting from their research, and how people are actually using these [2]. Many models have been suggested to explain the concept of research utilization in policymaking, some of which include frameworks for knowledge transfer as well as knowledge-driven, problem solving, interactive, enlightenment, and tactical models [3–6].

Research utilization can enhance policy decisions about resource allocation for services and programmes and decisions about how to deliver and finance those services [7, 8]. It can facilitate innovative changes that lead to improved client outcomes and promote critical thinking and reflective practice. In addition to ensuring that provision of safe and effective care practice is based on current, scientifically sound knowledge, effective research utilization validates researcher efforts, motivates scholars to continue to discover new knowledge, and reinforces professional accountability [9].

According to Weiss [10], research findings may be utilized in policymaking in three main forms: as data and findings, as ideas and criticism, or as briefs and arguments for action. Hanney et al. [4] argue that utilization of research findings in policymaking is sometimes instrumental, conceptual, or symbolic. Instrumental use involves the use of research findings directly in policy formulation; conceptual use refers to the gradual sedimentation of insight, theories, concepts, and perspectives; and symbolic use refers to use of research to support continuation of an already established position [4, 11]. Therefore, there are three phases of policymaking where research utilization might occur, namely agenda setting, policy formulation, and policy implementation [12].

Although previous studies have been conducted on health research utilization [13–20], there is limited data on how health research institutions promote utilization of their research findings to inform policy development and strategic planning. In striving to contribute towards improved health outcomes, health research institutions generate and accumulate huge volumes of potentially relevant results that remain largely underutilized, with limited movement from the research community to the practice community. Hanney et al. [4] note that, whereas “research centres undertake large scale dissemination, this does not necessarily guarantee that their research findings will be utilized”. This is further exacerbated by the fact that many dissemination practices remain based on “a mechanistic, linear conception of dissemination as a process of getting the word out” [2]. As a result, there is a fundamental gap between what is known about what works based on relevant knowledge and what is actually done with it; this is referred to as the “know-do gap” [21–24]. This know-do gap contributes to a relatively unchanged overall disease burden among low- and middle-income countries [15]. Collectively, these findings suggest that the concept of research utilization, including what is actually done to promote it, remains an area that is worth further consideration.

This study aims to (1) explore the activities implemented by researchers to promote research utilization and the extent to which they felt that their findings contributed to the policymaking process and (2) to document case studies of how research findings were actually utilized in the policymaking and strategic planning processes in Bangladesh. These study findings offer insights for a better understanding of the role that research institutions can play to promote utilization of their findings in policymaking and strategic planning processes.

## Methods

### Study design

**Fig. 1:**
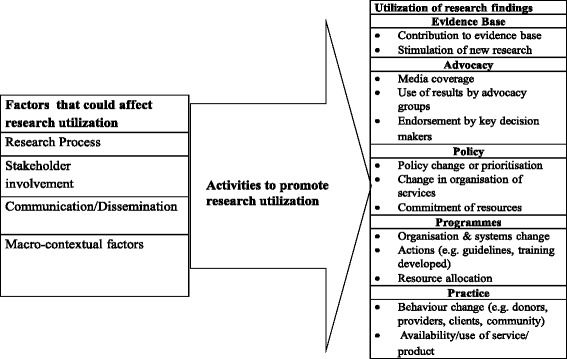
Adapted WHO/TRIP research utilization framework

The study used an exploratory case study design and relied on qualitative methods, including key informant interviews (KIIs), focus group discussions with study investigators, and database and document reviews. The study adapted the World Health Organization/Turning Research into Practice (WHO/TRIP) conceptual framework (Fig. 1) [25] to guide the documentation of case studies of research utilization in policymaking and strategic planning processes in Bangladesh. We conducted KIIs and focus group discussions with the International Centre for Diarrhoeal Disease Research, Bangladesh (icddr,b) researchers engaged in reproductive health-related research and policymakers, program implementers, and service providers involved in reproductive health research and service delivery within and immediately surrounding Dhaka, Bangladesh. A review of publications, documents and research databases was carried out for research studies registered and approved at icddr,b between 2001 and 2011. Data collection was conducted between September and December 2011.

### Study setting

The icddr,b, is an international health research institution, located in Dhaka, Bangladesh [26], dedicated to saving lives through research. It addresses a range of health areas, including child health, HIV/AIDs, communicable diseases, and reproductive health. In collaboration with academic and research institutions throughout the world, icddr,b conducts research, training and extension activities, as well as programme-based activities, to develop and share knowledge for global lifesaving solutions. Since 1978, icddr,b has shared its knowledge with the world, training more than 27,000 health professionals from over 78 countries.

### Study procedures

**Fig. 2:**
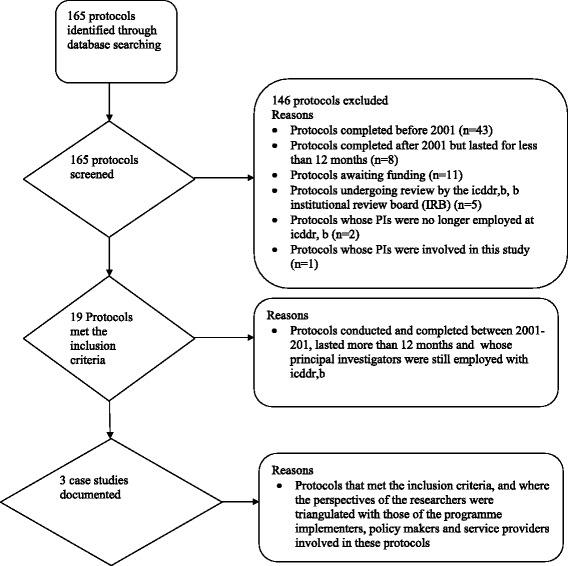
Study flow diagram

We used icddr,b’s database that tracks all approved study protocols carried out at icddr,b to search for research protocols of reproductive health studies conducted between 2001 and 2011 using the following search terms: ‘reproductive health’, ‘protocols’. This generated a list of 165 approved reproductive health research protocols conducted by icddr,b researchers. The protocols spanned a period of 18 years (1993–2011). These protocols were then screened using a checklist with an inclusion criteria focused on research that was conducted and completed between 2001 and 2011 as illustrated in Fig. 2. This time frame was picked based on the need to strike a balance between including protocols that were not too old to ensure we could interview the principal investigator (PI) or were not too recent allowing enough time for research findings to be utilized. Overall, 19 protocols met the inclusion criteria. Interviews were conducted with all researchers (n = 13) involved in the 19 selected protocols. Following the interviews with the study investigators, we established contact with the programme implementers who were involved in the implementation of these studies. A total of 15 KIIs were done (with researchers, programme implementers and policymakers) to explore a number of aspects relevant to research utilization, including how the research questions were identified; which stakeholders were involved at different stages of the research process; what communication and dissemination activities were undertaken by researchers; and what macro-contextual factors, such as the broader political, legal and programmatic climate, sensitivity of research topics, and cost considerations, may have impacted the utilization of the research findings. Three focus group discussions were also conducted to compare the perspectives of the researchers with those of the implementers and service providers involved in the implementation of the study protocols. All interviews (KIIs and focus group discussions) were conducted at convenient venues in the community, lasted between 30 and 90 minutes, and were audio-recorded after obtaining informed consent. We adapted the WHO/TRIP framework (Fig. 1) to examine the following factors that could have affected the utilization of study findings from the selected protocols, including the research process, stakeholder engagement, communication and dissemination, as well as macro contextual factors. Based on the WHO/TRIP framework and triangulation of information from the interviews, we documented three case studies in which research findings were utilized to inform policymaking and practice in Bangladesh.

### Data analysis

All qualitative data, including recordings were transcribed verbatim. Framework analysis [27], involving familiarization with data, development of a coding schedule, data coding, description of main themes, linking themes, and developing explanations of their relationship to each other was employed. Based on the codes, data were grouped into categories and thematic analysis was performed to facilitate identification of similar and divergent perspectives. The first author (DRW) undertook the coding of the interviews and read through them to ensure that all key themes were captured.

### Ethical considerations

Ethical approval and authorization to conduct the study was granted by the James P Grant School of Public Health Ethics Committee and the management of icddr,b. Informed consent was obtained from all respondents for their participation as well as permission to tape record all the interviews. Confidentiality and anonymity were assured for all participants, including their right to withdraw from the study at any time without offering a reason.

## Results

### Description of the reproductive health research protocols and participants’ characteristics

The 19 research protocols that met the inclusion criteria addressed five key thematic reproductive health areas, including sexually transmitted/reproductive tract infections (n = 6), maternal newborn and child health (n = 8), family planning (n = 2), adolescent and reproductive health (n = 2), and violence against women (n = 1). Nine protocols were led by three PIs (each had three protocols), four were led by two PI’s (each had two protocols), while six were led by seven different PIs. Out of the 21 participants interviewed for this study, four were female (three researchers, one programme implementer) and 17 were male (10 researchers, five programme implementers/service providers and two policymakers). The majority of the researchers had worked at icddr,b for over 5 years and held post-graduate qualifications in various disciplines. The response rate was 100 %.

### Activities undertaken to promote utilization of research findings

Twelve (92 %) out of the 13 researchers reported that they had conducted dissemination workshops, 7 (53 %) published scientific papers, 6 (46 %) developed policy briefs, 3 (23 %) provided technical assistance to policymakers, 3 (23 %) held one-on-one meetings with policymakers, 3 (23 %) produced reports, and 2 (15 %) engaged the media. All researchers reported engaging in several other activities to package their findings and to ensure that their key findings were picked up by their stakeholders. These included production of fact sheets, sharing findings on the website, engaging service providers, joining advocacy networks, and producing wind banners, among others:

“*We have fact sheets, one pager per topic, so that at a glance someone can get the messages. During disseminations we had face tools and wind banners with messages so that they are visible and they catch eyes. We had great media coverage, the dailies reported on this dissemination seminar and people got to know about some of these findings from there.*” (KII009)

“*One* [study] *was published in Social Science and Medicine and another one is in Sexually Transmitted Infections* [Journal] *and then in this field we also developed a review paper which was not only based on this study but rather partner notification in developing country settings. That was the kind of title that I published in BMC and we also developed a research brief from this partner notification study…*” (Focus Group Discussion 001)

When asked why they preferred the kind of activities they engaged in to facilitate the uptake of their research findings, researchers indicated that they based their decisions on the relative importance of each activity in terms of its ability to engage with the intended target audiences, impact and contribution to the researchers’ profile. One of the informants had this to say:

“*As you can see my 3 to 4 years previous works but you can't see the dissemination that I gave in the 3 years. And scientific papers obviously will have some scientific value for one’s career because these policy briefs have little weight on your career as a researcher…. These are not counted as scientific papers. So researchers are less interested in developing policy briefs, they would be more interested to write and publish papers.*” (KII002)

### Utilization of research findings

**Fig. 3:**
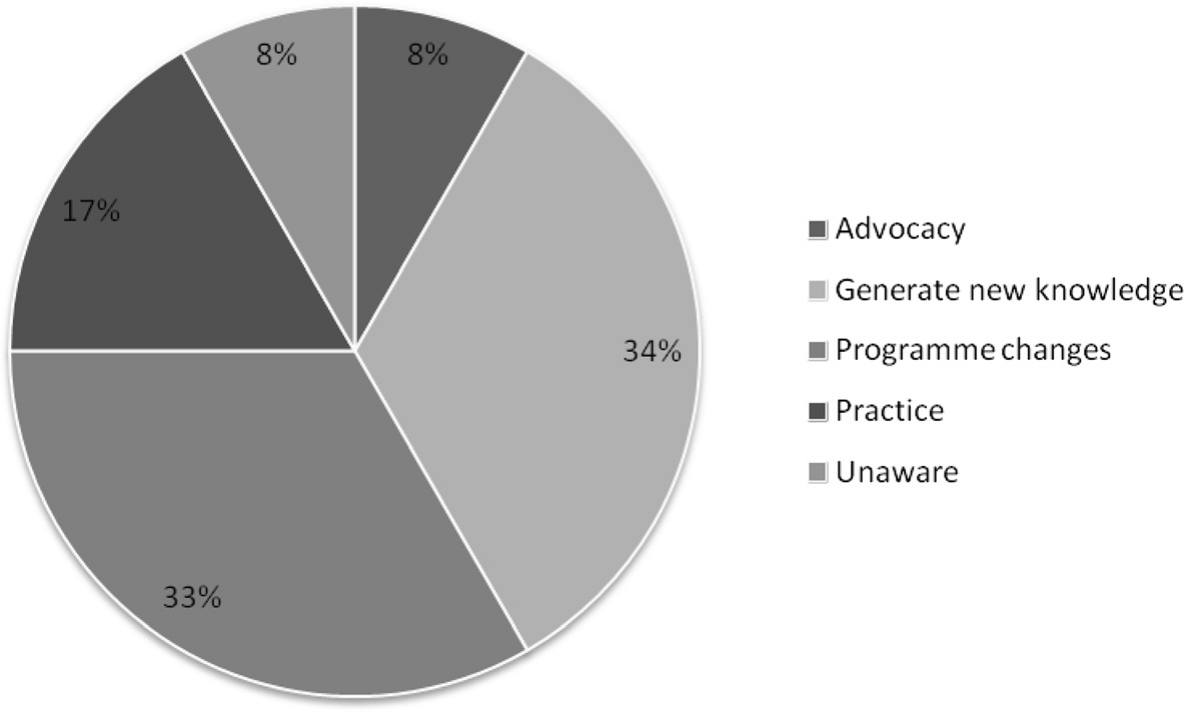
Perceptions of researchers regarding the utilization of their research findings

The majority of the researchers (12 out of 13) reported that findings from their studies were utilized and influenced policymaking processes at different levels. Fig. 3 shows the different ways in which researchers felt that findings from their studies were utilized. The researchers’ perspectives regarding the ways in which their findings were utilized were triangulated with responses from policymakers, programme implementers and service providers. Below are some of the responses from researchers, program implementers and service providers.

#### Scaling up interventions

One program implementer acknowledged the use of research findings from one of the studies to scale up the use of misoprostol for the prevention of postpartum haemorrhage (PPH) in Bangladesh [28].

“*… for the misoprostol one* [Scaling up the use of misoprostol for the prevention of postpartum haemorrhage study]*, findings were incorporated into the health system plans, both the operational one and the government has taken it up and has been scaling up in the 3 districts. So they have started the scaling up efforts in phases. They have also initiated the process to buy the tablet; they have allocated the money and have initiated the process to buy. Its moving it's not happening fast but it is happening.*” (KII012)

#### Generating new evidence and service delivery

Findings from two studies [29, 30] were reported to have instigated more research and hence contributed to generation of new evidence that influenced the decisions of program implementers to provide services to most at risk populations, including injecting drug users.

“*We just completed another study on injecting drug users and some of which was in that area and we are evaluating the ongoing outreach services and the services are being provided by Save the Children for the drug users*” (KII008)

The generation and subsequent use of new knowledge was reported to have influenced decisions that contributed to improved service delivery as noted by one of the program implementers in the quote below.

“*As a result of the* [Injecting Drug Users Dhaka City Study]*, service delivery was enhanced, we provided services in two shifts for seven days a week, so we intensified the programme and that has really helped.*” (KII013)

These findings suggest that research evidence was used at different levels of policymaking and implementation, including program implementation and further research. They also illustrate the co-production process in which researchers actively engaged and specifically sought the input of programme implementers to generate appropriate and relevant findings that facilitated the decision-making process.

### Case studies on processes of research utilization

Based on the WHO/TRIP research utilization framework, the study used the qualitative data collected to document three case studies of how research findings were utilized in the policymaking and strategic planning processes in Bangladesh. The three case studies were conducted between 2001 and 2008. Two of them used a quasi-experimental design and cohort study design, respectively, while one was a secondary analysis of longitudinal data from rural Bangladesh; they all reported policy-relevant findings [28, 30–35] (Table 1). For each case-study, we examined how the research questions were identified, which stakeholders were involved, the communication process, and the macro-contextual factors, as well as activities undertaken to ensure utilization of the findings.

**Table 1:** Characteristics and summary of the case studies of research utilization based on the WHO/TRIP framework

Case study 1. Scaling up the use of misoprostol for the prevention of postpartum hemorrhage in Bangladesh									
Characteristics of the study			Factors that could have contributed to research utilization				Activities to promote utilization	Utilization of research findings	Policy contribution
Study/Authors/years	Study design/ population	Main findings	Research Process (Problem identification)	Stakeholder involvement	Communication Process	Macro-contextual factors			
Md Abdul Quaiyum et al. 2014 [28]	Quasi experimental study design	The community acceptability of misoprostol tablets for the prevention of PPH reduced volume of blood loss after child birth	Literature review	Researchers/scientists of icddr,b	Dissemination workshops at international, national and community levels	Commitment from political parties	Seminars and dissemination workshops	Scaling up the use of misoprostol, and its inclusion into Essential Drug List (EDL) of Bangladesh	Ministry of Health and Family Welfare (MoHFW) and other key agencies committed to integrate misoprostol use into government policies and plans
	Women residing in the Abhoynagar sub district of Bangladesh	The delivery mat and pad were found to be useful for mothers as tools for assessing the amount of blood loss after delivery and informing care seeking decisions.	Conducted phased trial	Obstetric Gynecology Society of Bangladesh (OGSB)			Published in peer reviewed journals		Misoprostol was included in the program of activities for community health workers and family welfare agents
				Government Departments - DGHS, Drug Administration			Provided technical assistance		
				NGOs-BRAC, Pathfinder International, Engender Health, UN Agencies					
			*Researcher’s efforts to promote utilization of study findings*						
			*“We took a temporary permission from the Drug Administration of Bangladesh to conduct the research and subsequently, after we found that it is effective and useful for prevention of PPH and in collaboration with the drug companies and the pharmaceutical licensure of Bangladesh we requested them to extend the registration of the use of misoprostol for the prevention of PPH and we informed them that we will provide all the papers and any technical assistance they need and they did it and now misoprostol is registered in Bangladesh for use of in the prevention of PPH and that’s happened because of our research findings” (KII001)*						
Case study 2. Enactment of the Bangladesh Domestic Relations Bill 2010									
Characteristics of the study			Factors that could have contributed to research utilization				Activities to promote utilization	Utilization of research findings	Policy contribution
Study/Authors/years	Study design/ population	Main findings	Research Process (Problem identification)	Stakeholder involvement	Communication Process	Macro-contextual factors			
Asling-Monemi K, Tabassum Naved R, Perrsson LA, 2008 [31]	Secondary analysis of longitudinal data from rural Bangladesh of 2691 live-born children in relation to their mother's experience of physical, sexual and emotional partner violence and level of controlling behaviour in marriage.	Under five-mortality was 88 per 1000 in this cohort.	Conducted an exploratory study	Networks of women activists:	Dissemination seminars, Workshops	Political System is not gender sensitive	Published in peer reviewed journals	Advocacy	Research provided essential information to policy makers and human rights groups and reinforced the need for Bangladesh to introduce legislation to address domestic violence
		Severe physical violence and controlling behaviour in marriage were associated with higher under-five mortality among daughters of educated mothers in rural Bangladesh, indicating gender-based consequences of partner violence for child mortality.		Naripako, Citizen’s Initiative against Domestic Violence, Ministry of Women and Children Affairs	Published papers, Printed reports		Provided technical assistance	Enactment of Bangladesh DRB 2010	The parliament of Bangladesh passed the Domestic Violence (Protection and Prevention) Act 2010
							Joined advocacy network negotiated and lobbied		
							Engaged the media		
							Held one-on-one meetings		
							Produced wind banners		
			*Researcher’s efforts to promote utilization of study findings*						
			*“So what I was able to do was to sort of guide them in that, fine tuning the focus, and then provide input in defining different forms of violence which was not possible for them, because for the bill you have to get them pinned down. So that was the contribution from icddr,b based on our research”. (KII009)*						
Case study 3. Provision of outreach services to injecting drug users									
Characteristics of the study			Factors that could have contributed to research utilization				Activities to promote utilization	Utilization of research findings	Policy contribution
Study/Authors/years	Study design/ population	Main findings	Research Process (Problem identification)	Stakeholder involvement	Communication Process	Macro-contextual factors			
Tasnim Azim, Najmul Hussein and Robert Kelly [30]	Cohort study design	HIV prevalence still low in Bangladesh	Analysis of surveillance data	NGOs who were working with the drug users- CARE Bangladesh	Regular small meetings with the NGO	Harassment & violence against IDUs, Discrimination, Criminalization of IDUs	Held one on one meetings	Awareness creation among government officials, Provision of more services by NGOs	Strong government and NGO support for both serological and behavioral surveillance as demonstrated through the use of data to build national programmes
	Approximately 500 injecting Drug Users (IDUs) under CARE’s Needle/Syringe Exchange Programme (NEP)	Migration is a major source of new infections			Dissemination seminars		Published in peer reviewed journals	Advocacy for provision of oral substitution therapy	
		Deeply held cultural norms regarding acceptable behaviour, reluctance to use condoms and gender issues are major constrain to reducing the risk of an epidemic			Published in journals		Provided technical assistance		
		Interventions that are both effective and culturally acceptable to address the needs of the migrants are still absent.			Presentations at international and regional seminars.				
			*Researcher’s efforts to promote utilization of study findings*						
			*“As a result of this, we could also advocate for providing oral substitution therapy. .... the data from this study gave us the ammunition to go to the department of NARCOTICS Control that we definitely need to bring in oral drugs so that these people can stop injecting.” (KII008)*						

The research questions for the selected case studies were identified through a review of available literature, conducting exploratory case studies and analysis of secondary and surveillance data. Several stakeholders, including fellow researchers, professional organizations, non-governmental organizations (NGOs), civil society organizations, and government agencies, were involved during the research process in each of the three selected case studies. All researchers reported holding dissemination workshops involving the stakeholders described above. In addition to developing policy briefs and producing reports, they all reported publishing their findings in peer reviewed journals and providing technical assistance to the policymakers, programme implementers, and service providers. Two of the three researchers held one-on-one meetings with service providers and programme implementers, while one of them joined an advocacy network, produced wind banners and engaged the media.

When asked about the extra efforts researchers undertook to promote utilization of their findings in the policymaking process in Bangladesh, the researchers involved in the selected case studies reported having provided both research evidence and technical expertise to inform the drafting of policy:

“*We took a temporary permission from the Drug Administration of Bangladesh to conduct the research and subsequently, after we found that it is effective and useful for prevention of postpartum haemorrhage (PPH) and in collaboration with the drug companies and the Pharmaceutical Licensure of Bangladesh we requested them to extend the registration of the use of misoprostol for the prevention of PPH. We informed them that we will provide all the papers and any technical assistance you need and they did it and now misoprostol is registered in Bangladesh for use in the prevention of PPH and that’s happened because of our research findings*.” KII001

“*So what I was able to do was to sort of guide them in fine tuning the focus, and then provide input in defining different forms of violence which was not possible for them, because for the bill you have to get them* [forms of violence] *pinned down. So that was the contribution from icddr,b based on our research.*” KII009

However, some program implementers perceived inadequate efforts on the part of the institution icddr,b to engage the government in order to promote effective utilization of research findings.

“…*icddr,b if they want their results implemented in the public sector they should involve the government from the very beginning…maybe in a smaller role, the appropriate government person would be involved and that is lacking.*” (KII012)

Whereas the majority of the researchers reported about the activities and efforts undertaken to promote utilization of their research findings, only a few of them knew how and at what stages of the policymaking process their findings were utilized. For example, researchers reported that as a result of their constant engagement with policymakers and programme implementers, the Ministry of Health and Family Welfare and other key agencies committed to integrate misoprostol use into government policies and plans. Misoprostol was included in the program of activities for community health workers and family welfare agents as well as the Essential Drug List of Bangladesh. In the enactment of Bangladesh Domestic Bill 2010, upon conducting an exploratory study and understanding the magnitude of domestic violence in Bangladesh, researchers from icddr,b reported joining the Citizen’s Initiative against Domestic Violence (CIDV). This is a coalition of women’s organizations and human rights groups in Bangladesh that had already recognized the need for a legal framework to tackle domestic violence. However, they required technical input from researchers who, based on their research findings, supported CIDV to define the different forms of domestic violence and frame the challenge of domestic violence in Bangladesh. With this support, CIDV was able to lobby and engage the Ministry of Women and Children’s Affairs. As a result, both institutions refined the bill which was eventually placed on the agenda for the Bangladesh Parliament, which subsequently passed it into law.

## Discussion

Our study of the utilization of research findings to inform policy and practice in Bangladesh found the majority of researchers engaged in a variety of activities to inform the utilization process, including holding dissemination workshops, publishing in scientific journals, developing policy briefs, providing technical assistance, and holding one-on-one meetings with service providers and programme implementers. The majority of researchers also believed that their research findings had been utilized and had contributed towards influencing policy and practice within Bangladesh. Studies show that effective utilization of research findings can be enhanced through continuous stakeholder engagement before, during, and after the research process, making research findings more accessible, relevant, and easy to use, and with increased advocacy and communication efforts such as use of multiple channels to reach same audiences many times [8, 12, 20, 36, 37]. The researchers at icddr,b engaged in almost all these processes, which likely explains why their research findings were utilized in policymaking and strategic planning processes.

Dissemination seminars and scientific papers provide an opportunity for reaching out to the intended audiences with the message, and depict an attitude that has been described by the RUSH Project 2009 as “getting the word out” [2]. However, the limited number of researchers engaging in activities such as one-on-one meetings, media engagement, and advocacy highlights the limited efforts by researchers to strategically reach out to policymakers with their findings. Although budgetary and time constraints were cited as major limitations to engaging in such activities, promoting utilization of research findings requires explicit planning and resource allocation as well as monitoring and evaluation efforts [1, 25, 36, 37]. Additionally, the lack of incentives and institutionalized mechanisms by icddr,b to promote utilization of research findings hinders the research utilization process. To effectively contribute to the bridging of the know-do-gap, researchers need to strategically plan, allocate sufficient resources, and institute mechanisms to monitor and evaluate the utilization of their study findings. These strategic efforts should be institutionalized and implemented right from the pre-research, research and post-research stages of the research process.

Almost all researchers (12 out of 13) that reported utilization of evidence from their studies provided insights into the range of ways that research evidence can be utilized in the policymaking process. Results from the case studies show that research findings can be utilized as data and findings (instrumental), as ideas and criticism (conceptual), or as briefs and arguments for action (symbolic) [4, 10, 11]. For example, the enactment of the Bangladesh Domestic Relations Bill 2010 highlights the symbolic use of research findings. The input of the researchers helped to shape the advocacy efforts of the women networks which were then able to persuade the policymakers and legislators to eventually enact the Domestic Relations Bill 2010. Scaling up the use of misoprostol in the treatment of PPH points to the conceptual use of research evidence in policymaking. Knowledge generated from the study findings on assessing the feasibility, acceptability and program effectiveness of misoprostol in preventing PPH in rural Bangladesh helped to change the mindsets of the policymakers, programme implementers and service providers. The use of surveillance data in supporting decisions to provide outreach services to injecting drug users is an example of instrumental use of research findings in policymaking.

The results from this study also demonstrate that research findings can be utilized at different stages within the policymaking process, including during agenda setting, policy formulation and policy implementation [5, 12, 20]. For example, in the scaling up of the use of misoprostol and providing outreach services to injecting drug user case studies, findings were utilized at the policy implementation stage. Through the synthesis of available local and global evidence researchers were able to devise contextually acceptable interventions that not only contributed to accelerating the benefits of local innovation in strengthening the Bangladesh health system but also improved people’s health. In the enactment of Bangladesh Domestic Bill 2010, findings were effectively utilized at the agenda setting stage.

The engagement of several stakeholders, such as women’s advocacy networks, policymakers, NGOs and professional organizations, points to the on-going, iterative and complex nature of the policymaking process. It also emphasizes the reality that research findings are just one of the many ingredients that influence the process of policymaking [38]. Therefore, to effectively influence policymaking, it is important for researchers to acquaint themselves with the different stages and dynamics of the process. Familiarization with the policymaking process would not only enable researchers to engage the policymakers at the right time but also to identify which stage of the process they wish to or are most likely to influence with their results; this would also provide insights into which strategies and tools to use during these engagements.

There are various ways through which research findings can be utilized to inform the policymaking process, including documentation, analysis and prescription [12]. However, determining the contribution of a particular piece of research in the development of certain policies remains a challenge [20]. As such, a key limitation of this study was that few research end users were interviewed, which made triangulation of information collected from the researchers involved in the 19 studies rather challenging. Nonetheless, the three selected case studies provided additional insight into the activities, efforts to promote, and actual utilization of research findings into policy and practice. Future research could, however, benefit from employing methods to verify perceived impacts of research. Additionally, there may also have been some recall bias due to the duration between completion of the studies and the time this study was conducted. However, the engagement of the researchers, programme implementers and service providers that were directly involved in these studies gives credence to the study findings as these provided first-hand information and experiences.

Effective and timely research utilization has the potential to facilitate innovative changes that can lead to improved client outcomes and promotes critical thinking and reflective practice. In addition to ensuring that provision of safe and effective healthcare practice is based on current, scientifically sound knowledge, research utilization validates the efforts of the researcher, motivates scholars to continue to discover new knowledge, and reinforces professional accountability [9]. These benefits can only be accrued if health research organizations, such as icddr,b, can devise and implement strategic efforts to promote the utilization of research findings in policymaking and strategic planning processes.

## Conclusions

The study results show that active and continuous stakeholder engagement through multiple channels of communication is critical to ensuring utilization of research findings in policymaking. Additionally, promoting research utilization requires paying attention to the micro- and macro-contextual factors such as the existence of global policies that support accelerated uptake of locally generated evidence.

## Abbreviations

CIDV: Citizen’s Initiative against Domestic Violence
icddr,b: International Centre for Diarrhoeal Disease Research, Bangladesh
KIIs: Key informant interviews
NGOs: Non-governmental organizations
PI: Principal investigator
PPH: Prevention of postpartum haemorrhage
WHO/TRIP: World Health Organization/Turning Research into Practice
